# Supplementary material for: Prefrontal cortical dynorphin peptidergic transmission constrains threat-driven behavioral and network states
Source: bioRxiv. 2024 Jan 9:2024.01.08.574700. Preprint. [Version 1] doi: 10.1101/2024.01.08.574700 (PMC10822088; doi:10.1101/2024.01.08.574700)
Supplement: Supplement 1 [file media-1.pdf]

Table 1

Figure	Sub-figure	Statistical Test	One tailed or two tailed?	t, D, or F value	P value	If there is signifance, where the signifciance occurred (multiple comparison)?
**Fig.1B**	**Freezing in conditioning**	RM two-way ANOVA with Bonferroni's Post Hoc test		Group Main Effect, F (1, 13) = 99.65	p<0.0001	Difference between tone only and tone + footshock group from Tone 3 to Tone10
				Trial Main Effect, F (9, 117) = 9.665	p<0.0001	
				Group x Trial interaction, F (9, 117) = 6.607	p<0.0001	Difference between tone only and tone + footshock mice during specific epochs
**Fig.1C**	**Freezing in exctinction**	RM two-way ANOVA with Bonferroni's Post Hoc test		Group Main Effect, F (1, 13) = 39.92	p<0.0001	Difference between tone only and tone + footshock group in all trials
				Trial Main Effect, F (9, 117) = 0.9754	p=0.4641	
				Group x Trial interaction, F (9, 117) = 0.7390	p=0.6725	
**Fig.1D**	**Conditioning day 1**	RM two-way ANOVA with Bonferroni's Post Hoc test		Group Main Effect, F (1, 13) = 43.05	P<0.0001	Difference between tone only and tone + footshock mice
				Time Main Effect, F (560, 7280) = 8.380	P<0.0001	
				Group x Time interaction, F (560, 7280) = 10.86	p<0.0001	Difference between tone only and tone + footshock mice during specific epochs
**Fig.1D**	**Conditioning day 3**	RM two-way ANOVA with Bonferroni's Post Hoc test		Group Main Effect, F(1,13)=11.12	P=0.0054	Difference between tone only and tone + footshock mice
				Time Main Effect, F (560, 7280) = 5.692	p<0.0001	
				Group x Time interaction, F (560, 7280) = 5.014	p<0.0001	Difference between tone only and tone + footshock mice during specific epochs
**Fig.1D**	**Extinction**	RM two-way ANOVA		Group Main Effect, F(1,13)=0.3375	p=0.5712	
				Time Main Effect, F(560,7280)=0.5750	p>0.9999	
				Group x Time interaction, F(560,7280)=0.4342	p>0.9999	
**Fig.1E**	**AUC in conditioning day 1&3**	RM three-way ANOVA with Bonferroni's Post Hoc test		Group Main Effect, F (1, 13) = 15.78	p=0.0016	Tone 1-3:tone+footshock day3 vs. Tone 1-3:tone only day1 (p=0.0017); Tone 1-3:tone+footshock day3 vs. Tone 1-3:tone only day3 (p=0.0389);
				Days Main Effect, F (1, 13) = 4.180	p=0.0617	
				Trial Main Effect, F (2, 26) = 0.09033	p=0.9139	
**Fig.1E**	**AUC in extinction day 1**	RM two-way ANOVA		Group Main Effect, F (1, 13) = 0.3314	p=0.5747	
				Trial Main Effect, F (9, 117) = 1.737	P=0.0880	
				Group x Trial interaction, F (9, 117) = 1.453	P=0.1736	
**Fig.1I**		RM wo-way ANOVA with Bonferroni's Post Hoc test		Cell Main Effect, F (1, 10) = 1.965	p=0.1912	
				Trial Main Effect, F (5, 50) = 2.854	p=0.0241	
				Cell x Trial interaction, F (5, 50) = 3.811	p=0.0053	Footshock 1: WT vs. PDyn-Cre (p=0.0019)
**Fig.1J**	**Footshock 1**	RM two-way ANOVA with Bonferroni's Post Hoc test		Cell Main Effect, F (1, 2184) = 184.2	P<0.0001	WT vs. PDyn-Cre: significant different from 1s to 7s.
				Time Main Effect, F (23, 2184) = 14.93	P<0.0001	
				Cell x Time interaction, F (23, 2184) = 7.227	P<0.0001	
**Fig.1J**	**Footshock 6**	RM two-way ANOVA with Bonferroni's Post Hoc test		Cell Main Effect, F (1, 1848) = 89.13	P<0.0001	WT vs. PDyn-Cre: significant different from 3.5s to 7s.
				Time Main Effect, F (23, 1848) = 30.45	P<0.0001	
				Cell x Time interaction, F (23, 1848) = 6.980	P<0.0001	
**Fig.1K**		RM two-way ANOVA with Bonferroni's Post Hoc test		Cell Main Effect, F (1, 510) = 75.33	p<0.0001	Footshock 1: WT vs. PDyn-Cre (p<0.0001); Footshock 2: WT vs. PDyn-Cre (p=0.0002); Footshock 3: WT vs. PDyn-Cre (p=0.0098); Footshock 4: WT vs. PDyn-Cre (p=0.0309)
				Trial Main Effect, F (5, 510) = 6.484	p<0.0001	
				Cell x Trial interaction, F (5, 510) = 4.907	p=0.0002	
**Fig.1L**		Paired t-test	two tailed	t=2.785	p=0.0496	
**Fig.1M**		RM two-way ANOVA		Cell Main Effect, F (1, 10) = 4.018	p=0.0728	
				Trial Main Effect, F (5, 50) = 1.284	p=0.2854	
				Cell x Trial interaction, F (5, 50) = 1.761	p=0.1382	

Figure	Sub-figure	Statistical Test	One tailed or two tailed?	t, D, or F value	P value	If there is signifance, where the signifciance occurred (multiple comparison)?
**Fig.1N**	**Tone 1**	RM two-way ANOVA with Bonferroni's Post Hoc test		Cell Main Effect, F (1, 4320) = 240.9	P<0.0001	WT vs. PDyn-Cre: significant different from 32s to 35s.
				Time Main Effect, F (79, 4320) = 1.706	P=0.0001	
				Cell x Time interaction, F (79, 4320) = 1.487	P=0.0036	
**Fig.1N**	**Tone 6**	RM two-way ANOVA		Cell Main Effect, F (1, 6400) = 7.352e-030	P>0.9999	
				Time Main Effect, F (79, 6400) = 1.523	P=0.0021	
				Cell x Time interaction, F (79, 6400) = 9.307e-032	P>0.9999	
**Fig.1O**		RM two-way ANOVA with Bonferroni's Post Hoc test		Cell Main Effect, F (1, 360) = 13.91	p=0.0002	**Tone 3:** WT vs. PDyn-Cre (p=0.0074)
				Trial Main Effect, F (5, 360) = 2.856	p=0.0152	
				Cell x Trial interaction, F (5, 360) = 1.678	p=0.139	
**Fig.1P**		Paired t-test	two tailed	t=2.246	p=0.0880	
**Fig.1Q**		Unpaired t-test	two tailed	t=1.260	p=0.2364	
**Fig.1S**		Chi-square test		Chi-square=4.342, df=8	p=0.8251	
**Fig. 1T**		RM two-way ANOVA with Bonferroni's Post Hoc test		Cell Main Effect, F (1, 155) = 2.328	p=0.1291	**Speed bin 48:** WT vs. PDyn-Cre (p=0.0382)
				Speed Main Effect, F (16, 2480) = 4.088	p<0.0001	
				Cell x Speed interaction, F (16, 2480) = 1.720	p=0.0367	
**Fig.2D**		Two-way ANOVA with Bonferroni's Post Hoc test		Sub-region Main Effect, F (1, 12) = 4.460e-013	P>0.9999	
				Cell-Type Main Effect, F (1, 12) = 1011	P<0.0001	**PrL:**VGluT1 vs. PrL:VGAT (P<0.0001); **IL:**VGluT1 vs. IL:VGAT (P<0.0001);
				Sub-region x Cell-Type interaction, F (1, 12) = 2.746	P=0.1234	
**Fig.2F**		Unpaired t-test	two tailed	t=4.740	p=0.0032	
**Fig.2G**		Kolmogorov-Smirnov test		D=0.2677	p<0.0001	
**Fig.2I**		Unpaired t-test	two tailed	t=3.513	P=0.0007	
**Fig.2N**		RM two-way ANOVA		AP frequency Main Effect, F (12, 234) = 8.509	P<0.0001	
				Cell-Type Main Effect, F (1, 234) = 13.89	P=0.0002	
				Sub-region x Cell-Type interaction, F (12, 234) = 0.2115	P=0.9979	
**Fig.3J**	**Tone (0-10s)**	RM two-way ANOVA with Bonferroni's Post Hoc test		Days Main Effect, F (1, 18) = 28.80	P<0.0001	
				Cell-type Main Effect, F (2, 18) = 8.611	P=0.0024	**Conditioning day1:** Con/Foff vs. Con/Fon (p = 0.0043); Con/Foff vs. Coff/Fon (p = 0.0001)
				Days x Cell-type interaction, F (2, 18) = 4.623	P=0.0240	
**Fig.3J**	**Tone (10-28s)**	RM two-way ANOVA with Bonferroni's Post Hoc test		Days Main Effect, F (1, 18) = 17.13	P=0.0006	
				Cell-type Main Effect, F (2, 18) = 5.823	P=0.0112	**Conditioning day1:** Con/Foff vs. Con/Fon (p = 0.0008); Con/Foff vs. Coff/Fon (p = 0.0121). **Extinction day1:** Con/Fon vs. Coff/Fon (p = 0.0242)
				Days x Cell-type interaction, F (2, 18) = 8.311	P=0.0028	
**Fig.3J**	**Footshock (28-33s)**	One-way ANOVA with Bonferroni's Post Hoc test		F (2, 19) = 1.551	P=0.2377	
**Fig.3J**	**Post-footshock (33-50s)**	One-way ANOVA with Bonferroni's Post Hoc test		F (2, 19) = 13.48	P=0.0002	Con/Foff vs. Con/Fon (p = 0.0002); Con/Fon vs. Coff/Fon (p = 0.0192)
**Fig.4D**		RM two-way ANOVA		Time Main Effect, F(2399,14394)=9.426	p<0.0001	
				Treatment Main Effect, F(1,6)=10.98	p=0.0161	
				Time x Treatment interaction, F(2399,14394)=11.57	p<0.0001	
**Fig.4K**		RM two-way ANOVA with Bonferroni's Post Hoc test		Treatment Main Effect, F (1, 16) = 7.690	P=0.0136	**kLight 1.2a:** Tone control vs. Conditioning (p = 0.0046)
				Sensor Main Effect, F (1, 16) = 3.824	P=0.0682	**Conditioning:** kLight1.2 vs. kLight0 (P = 0.034)
				Treatment x Sensor interaction, F (1, 16) = 3.277	P=0.0891	

Figure	Sub-figure	Statistical Test	One tailed or two tailed?	t, D, or F value	P value	If there is signifance, where the significiance occurred (multiple comparison)?
**Fig.4M**	**Number of Events**	RM two-way ANOVA with Bonferroni's Post Hoc test		Trial Main Effect, F (1, 8) = 19.30	P=0.0023	**kLight 1.2a:** Baseline vs. Restrained (p = 0.001)
				Sensor Main Effect, F (1, 8) = 7.292	P=0.0271	**Restrained:** kLight1.2 vs. kLight0 (P = 0.003)
				Trial x Sensor interaction, F (1, 8) = 7.292	P=0.0271	
**Fig.4M**	**AUC**	RM two-way ANOVA with Bonferroni's Post Hoc test		Trial Main Effect, F (1, 8) = 3.083	P=0.1172	**kLight 1.2a:** Baseline vs. Restrained (p = 0.0087)
				Sensor Main Effect, F (1, 8) = 11.37	P=0.0097	**Restrained:** kLight1.2 vs. kLight0 (P = 0.0005)
				Trial x Sensor interaction, F (1, 8) = 10.32	P=0.0124	
**Fig.5B**	**Z-Score**	RM two-way ANOVA with Bonferroni's Post Hoc test		shRNA Main Effect, F(1, 9) = 0.03346	P=0.8589	
				Time Main Effect, F (560.0, 5040) = 4.996	p<0.0001	
				shRNA x Time interaction,F (560, 5040) = 2.245	p<0.0001	
**Fig.5C**	**Z-Score**	RM two-way ANOVA with Bonferroni's Post Hoc test		shRNA Main Effect, F (1, 8) = 0.2007	P=0.6660	
				Time Main Effect, F (560.0, 4480) = 4.429	p<0.0001	
				shRNA x Time interaction,F (560, 4480) = 2.669	p<0.0001	
**Fig.5D**	**Conditioning**	RM two-way ANOVA		shRNA Main Effect, F (1, 9) = 2.360	P=0.1589	
				Trial Main Effect, F (4, 36) = 0.7895	P=0.5397	
				shRNA x Trial interaction, F (4, 36) = 0.5907	P=0.6715	
**Fig.5D**	**Extinction**	RM two-way ANOVA		shRNA Main Effect, F (1, 9) =0.05216	P=0.8245	
				Trial Main Effect, F (9, 81) = 1.898	P=0.0638	
				shRNA x Trial interaction, F (9, 81) = 3.504	P=0.0011	**Tone 2:** shCtrl vs shPDyn (p=0.0012)
**Fig.5F**	**Mean Intensity**	Unpaired t-test	two tailed	t=4.582	P=0.0010	
**Fig.5F**	**# of cells / mm^2**	Unpaired t-test	two tailed	t=4.459	p=0.0012	
**Fig.5H**	**Tone**	RM two-way ANOVA with Bonferroni's Post Hoc test		Trial Main Effect, F (5, 105) = 51.75	P<0.0001	
				shRNA Main Effect, F (1, 21) = 2.828	P=0.1075	
				Trial x shRNA interaction, F (5, 105) = 2.759	P=0.0221	Tone3:shCtrl vs. shPDyn (P=0.0357)
**Fig.5H**	**ITI**	RM two-way ANOVA with Bonferroni's Post Hoc test		Trial Main Effect, F (5, 105) = 63.82	P<0.0001	
				shRNA Main Effect, F (1, 21) = 12.82	P=0.0018	Tone2:shCtrl vs. shPDyn (P=0.0157); Tone3:shCtrl vs. shPDyn (P=0.0082); Tone5:shCtrl vs. shPDyn (P=0.0013)
				Trial x shRNA interaction, F (5, 105) = 2.125	P=0.0682	
**Fig.5J**	**CS+**	RM two-way ANOVA		Trial Main Effect, F (9, 198) = 28.20	P<0.0001	
				shRNA Main Effect, F (1, 22) = 2.249	P=0.1479	
				Trial x shRNA interaction, F (9, 198) = 1.157	P=0.3245	
**Fig.5J**	**CS-**	RM two-way ANOVA with Bonferroni's Post Hoc test		Trial Main Effect, F (9, 198) = 20.42	P<0.0001	
				shRNA Main Effect, F (1, 22) = 6.453	P=0.0187	**Trial7**:shCtrl vs. shPDyn (P=0.0229); **Trial9:**shCtrl vs. shPDyn (P=0.0483); **Trial10:**shCtrl vs. shPDyn (P=0.0452)
				Trial x shRNA interaction, F (9, 198) = 2.928	P=0.0028	
**Fig.5K**		Unpaired t-test	two tailed	t=2.473	p=0.0216	
**Fig.6C**		Kolmogorov-Smirnov test		D=0.001389	P>0.9999	
**Fig.6E**	**Tone**	Kolmogorov-Smirnov test		D=0.04697	P=0.3108	
**Fig.6E**	**Footshock**	Kolmogorov-Smirnov test		D=0.09809	P=0.0006	
**Fig.6E**	**ITI**	Kolmogorov-Smirnov test		D=0.1187	P<0.0001	
**Fig.6G**	**Tone**	Kolmogorov-Smirnov test		D=0.07091	P=0.0290	
**Fig.6G**	**Footshock**	Kolmogorov-Smirnov test		D=0.08962	P=0.0023	
**Fig.6G**	**ITI**	Kolmogorov-Smirnov test		D=0.1141	P<0.0001	

Figure	Sub-figure	Statistical Test	One tailed or two tailed?	t, D, or F value	P value	If there is signifance, where the signifciance occurred (multiple comparison)?
**Fig.6H**		Two-way ANOVA		Trial Main Effect, F (5, 45) = 4.294	P=0.0028	
				shRNA Main Effect, F (1, 9) = 0.5528	P=0.4761	
				Trial x shRNA interaction, F (5, 45) = 1.025	P=0.4143	
**Fig.6J**		Two-way ANOVA		Trial Main Effect, F (5, 894) = 0.2352	P=0.9471	
				shRNA Main Effect, F (1, 894) = 3.210	P=0.0735	
				Trial x shRNA interaction, F (5, 894) = 0.1597	P=0.9771	
**Fig.6K**		Two-way ANOVA		Trial Main Effect, F (5, 45) = 3.767	P=0.0062	
				shRNA Main Effect, F (1, 9) = 0.2084	P=0.6588	
				Trial x shRNA interaction, F (5, 45) = 0.6519	P=0.6615	
**Fig.6M**		Two-way ANOVA		Trial Main Effect, F (5, 808) = 0.5240	P=0.7583	
				shRNA Main Effect, F (1, 808) = 0.4076	P=0.5234	
				Trial x shRNA interaction, F (5, 808) = 0.8868	P=0.4893	
**Fig.6N**		Two-way ANOVA		Trial Main Effect, F (5, 45) = 3.525	P=0.0090	
				shRNA Main Effect, F (1, 9) = 3.450	P=0.0962	
				Trial x shRNA interaction, F (5, 45) = 0.7599	P=0.5835	
**Fig.6P**		Two-way ANOVA		Trial Main Effect, F (5, 745) = 0.2647	P=0.9323	
				shRNA Main Effect, F (1, 745) = 2.122	P=0.1456	
				Trial x shRNA interaction, F (5, 745) = 2.380	P=0.0372	
**Fig.6Q**		Unpaired t-test	two tailed	t=2.255	p=0.0506	
**Fig.6S**		Chi-square test		Chi-square=10.99, df=7	p=0.1392	
**Fig.6T**		Two-way ANOVA with Bonferroni's Post Hoc test		Speed Main Effect, F (16, 5696) = 10.28	P<0.0001	
				shRNA Main Effect, F (1, 356) = 1.337	P=0.2484	
				Speed x shRNA interaction, F (16, 5696) = 2.061	P=0.0076	
**Fig.7B**		RM two-way ANOVA with Bonferroni's Post Hoc test		Trial Main Effect, F (5, 30) = 5.566	P=0.0010	
				shRNA Main Effect, F (1, 6) = 0.3320	P=0.5855	
				Trial x shRNA interaction, F (5, 30) = 1.047	P=0.4087	
**Fig.7C**		RM two-way ANOVA with Bonferroni's Post Hoc test		Trial Main Effect, F (5, 30) = 6.156	P=0.0005	
				shRNA Main Effect, F (1, 6) = 6.828	P=0.0400	
				Trial x shRNA interaction, F (5, 30) = 1.040	P=0.4124	
**Fig.7D**		RM two-way ANOVA with Bonferroni's Post Hoc test		Trial Main Effect, F (5, 30) = 2.758	P=0.0364	
				shRNA Main Effect, F (1, 6) = 10.82	P=0.0166	shCtrl_trial3 vs. shPDyn_trial3 (P=0.0250); shCtrl_trial5 vs. shPDyn_trial5 (P=0.0176)
				Trial x shRNA interaction, F (5, 30) = 0.5040	P=0.7708	
**Fig.7E**		RM two-way ANOVA with Bonferroni's Post Hoc test		Trial Main Effect, F (5, 30) = 40.05	P<0.0001	
				shRNA Main Effect, F (1, 6) = 0.1791	P=0.6869	
				Trial x shRNA interaction, F (5, 30) = 0.4782	P=0.7896	
**Fig.7F**		RM two-way ANOVA with Bonferroni's Post Hoc test		Trial Main Effect, F (5, 30) = 12.02	P<0.0001	
				shRNA Main Effect, F (1, 6) = 0.1079	P=0.7537	
				Trial x shRNA interaction, F (5, 30) = 0.4053	P=0.8413	

Figure	Sub-figure	Statistical Test	One tailed or two tailed?	t, D, or F value	P value	If there is signifance, where the signifciance occurred (multiple comparison)?
**Fig.7G**		RM two-way ANOVA with Bonferroni's Post Hoc test		Trial Main Effect, F (5, 30) = 27.19	P<0.0001	
				shRNA Main Effect, F (1, 6) = 9.971	P=0.0196	shCtrl_trial2 vs. shPDyn_trial2 (P=0.0255); shCtrl_trial3 vs. shPDyn_trial3 (P=0.0046); shCtrl_trial6 vs. shPDyn_trial6 (P=0.0367)
				Trial x shRNA interaction, F (5, 30) = 1.070	P=0.3965	
**Fig.7I**		Unpaired t-test	two tailed	t=1.817	P=0.1027	
**Fig.7J**		Two-way ANOVA with Bonferroni's Post Hoc test		Data Main Effect, F (1, 18) = 53.86	P<0.0001	**shCtrl:** Actural data vs. Shuffled data (P<0.0001);**shPDyn:** Actural data vs. Suffled data (P=0.0013)
				shRNA Main Effect, F (1, 18) = 3.197	P=0.0906	**Actural data:** shCtrl vs. shPDyn (p=0.0419)
				Data x shRNA interaction, F (1, 18) = 3.206	P=0.0902	
**Supp Fig.1A**	**Conditioning day1, Tone**	RM two-way ANOVA with Bonferroni's Post Hoc test		Group Main Effect, F (1, 13) = 99.65	p<0.0001	Difference between tone only and tone + footshock group from Tone 3 to Tone10
				Trial Main Effect, F (9, 117) = 9.665	p<0.0001	
				Group x Trial interaction, F (9, 117) = 6.607	p<0.0001	
**Supp Fig.1A**	**Conditioning day2, Tone**	RM two-way ANOVA with Bonferroni's Post Hoc test		Group Main Effect, F (1, 13) = 53.64	p<0.0001	Difference between tone only and tone + footshock group in all trials
				Trial Main Effect, F (9, 117) = 0.4923	p=0.8773	
				Group x Trial interaction, F (9, 117) = 0.5008	p=0.8714	
**Supp Fig.1A**	**Conditioning day3, Tone**	RM two-way ANOVA with Bonferroni's Post Hoc test		Group Main Effect, F (1, 13) = 25.87	p=0.0002	Difference between tone only and tone + footshock group in all trials
				Trial Main Effect, F (9, 117) = 1.321	p=0.2334	
				Group x Trial interaction, F (9, 117) = 1.515	p=0.1505	
**Supp Fig.1A**	**Extinction day1, Tone**	RM two-way ANOVA with Bonferroni's Post Hoc test		Group Main Effect, F (1, 13) = 39.92	p<0.0001	Difference between tone only and tone + footshock group in all trials
				Trial Main Effect, F (9, 117) = 0.9754	p=0.4641	
				Group x Trial interaction, F (9, 117) = 0.7390	p=0.6725	
**Supp Fig.1A**	**Extinction day2, Tone**	RM two-way ANOVA with Bonferroni's Post Hoc test		Group Main Effect, F (1, 13) = 26.25	p=0.0002	Difference between tone only and tone + footshock group in all trials
				Trial Main Effect, F (9, 117) = 1.246	p=0.2742	
				Group x Trial interaction, F (9, 117) = 1.286	p=0.2517	
**Supp Fig.1A**	**Renewal, Tone**	RM two-way ANOVA with Bonferroni's Post Hoc test		Group Main Effect, F (1, 13) = 5.048	p=0.0427	Tone1: Tone only vs. Tone+footshock (p=0.0137)
				Trial Main Effect, F (4, 52) = 1.707	p=0.1625	
				Group x Trial interaction, F (4, 52) = 1.867	p=0.1303	
**Supp Fig.1A**	**Conditioning day1, ITI**	RM two-way ANOVA with Bonferroni's Post Hoc test		Group Main Effect, F (1, 13) = 64.64	p<0.0001	Difference between tone only and tone + footshock group from ITI 3 to ITI 10
				Trial Main Effect, F (9, 117) = 6.319	p<0.0001	
				Group x Trial interaction, F (9, 117) = 3.856	p=0.0003	
**Supp Fig.1A**	**Conditioning day2, ITI**	RM two-way ANOVA with Bonferroni's Post Hoc test		Group Main Effect, F (1, 13) = 105.1	p<0.0001	Difference between tone only and tone + footshock group in all trials
				Trial Main Effect, F (9, 117) = 2.115	p=0.0335	
				Group x Trial interaction, F (9, 117) = 1.482	p=0.1626	
**Supp Fig.1A**	**Conditioning day3, ITI**	RM two-way ANOVA with Bonferroni's Post Hoc test		Group Main Effect, F (1, 13) = 81.16	p<0.0001	Difference between tone only and tone + footshock group in all trials
				Trial Main Effect, F (9, 117) = 1.048	p=0.4069	
				Group x Trial interaction, F (9, 117) = 0.7321	p=0.6788	

Figure	Sub-figure	Statistical Test	One tailed or two tailed?	t, D, or F value	P value	If there is signifance, where the signifciance occurred (multiple comparison)?
**Supp Fig.1A**	**Extinction day1, ITI**	RM two-way ANOVA with Bonferroni's Post Hoc test		Group Main Effect, F (1, 13) = 15.18	p=0.0018	Difference between tone only and tone + footshock group in ITI2, ITI4, ITI10, ITI12, ITI14, and ITI16
				Trial Main Effect, F (9, 117) = 1.098	p=0.3699	
				Group x Trial interaction, F (9, 117) = 1.190	p=0.3077	
**Supp Fig.1A**	**Extinction day2, ITI**	RM two-way ANOVA with Bonferroni's Post Hoc test		Group Main Effect, F (1, 13) = 16.80	p=0.0013	Difference between tone only and tone + footshock group in ITI2, ITI4, ITI10, and ITI12
				Trial Main Effect, F (9, 117) = 0.7721	p=0.6424	
				Group x Trial interaction, F (9, 117) = 0.5106	p=0.8645	
**Supp Fig.1A**	**Renewal, ITI**	RM two-way ANOVA with Bonferroni's Post Hoc test		Group Main Effect, F (1, 13) = 2.535	p=0.1354	
				Trial Main Effect, F (4, 52) = 0.1675	p=0.9539	
				Group x Trial interaction, F (4, 52) = 0.4318	p=0.7850	
**Supp Fig.1B**	**Conditioning day2**	RM two-way ANOVA with Bonferroni's Post Hoc test		Group Main Effect, F (1, 13) = 51.86	P<0.0001	Difference between tone only and tone + footshock mice in multiple epochs
				Time Main Effect, F (560, 7280) = 8.394	P<0.0001	
				Group x Time interaction, F (560, 7280) = 9.578	p<0.0001	
**Supp Fig.1B**	**Extinction day2**	RM two-way ANOVA		Group Main Effect, F (1, 13) = 0.4369	P=0.5202	
				Time Main Effect, F (560, 7280) = 0.5223	P>0.9999	
				Group x Time interaction, F (560, 7280) = 0.5495	P>0.9999	
**Supp Fig.1B**	**Renewal**	RM two-way ANOVA		Group Main Effect, F (1, 13) = 1.473	P=0.2464	
				Time Main Effect, F (560, 7280) = 0.6172	P>0.9999	
				Group x Time interaction, F (560, 7280) = 0.9297	p=0.8741	
**Supp Fig.1C**		RM two-way ANOVA		Group Main Effect, F (1, 13) = 19.98	P=0.0006	Day1: Tone only vs. Tone+footshock (p=0.0075); Day2: Tone only vs. Tone+footshock (p=0.0005); Day3: Tone only vs. Tone+footshock (p=0.0009)
				Days Main Effect, F (2, 26) = 3.190	P=0.0577	
				Group x Days interaction, F (2, 26) = 0.5919	P=0.5605	
**Supp Fig.1D**		RM two-way ANOVA		Group Main Effect, F (1, 13) = 0.5352	P=0.4774	
				Days Main Effect, F (1, 13) = 0.1050	P=0.7511	
				Group x Days interaction, F (1, 13) = 0.09517	P=0.7626	
**Supp Fig.1E**		Unpaired t-test	two tailed	t=1.257	P=0.2309	
**Supp Fig.1F**		RM two-way ANOVA		Cell Main Effect, F (1, 10) = 0.2615	p=0.6202	
				Trial Main Effect, F (5, 50) = 0.5202	p=0.7598	
				Cell x Trial interaction, F (5, 50) = 1.573	p=0.1848	
**Supp Fig.1G**	**ITI 1**	RM two-way ANOVA		Cell Main Effect, F (1, 6890) = 0.2456	P=0.6202	
				Time Main Effect, F (129, 6890) = 0.9264	P=0.7124	
				Cell x Time interaction, F (129, 6890) = 0.2632	P>0.9999	
**Supp Fig.1G**	**ITI 6**	RM two-way ANOVA with Bonferroni's Post Hoc test		Cell Main Effect, F (1, 6853) = 41.72	P<0.0001	
				Time Main Effect, F (129, 6853) = 2.899	P<0.0001	
				Cell x Time interaction, F (129, 6853) = 0.3288	P>0.9999	
**Supp Fig.1H**		RM two-way ANOVA		Cell Main Effect, F (1, 328) = 3.227	p=0.0734	
				Trial Main Effect, F (5, 328) = 0.9110	p=0.474	
				Cell x Trial interaction, F (5, 328) = 0.7953	p=0.5536	

Figure	Sub-figure	Statistical Test	One tailed or two tailed?	t, D, or F value	P value	If there is signifance, where the signifciance occurred (multiple comparison)?
**Supp Fig.1I**		Paired t-test	two tailed	t=0.4533	p=0.6738	
**Supp Fig.2A**		Unpaired t-test	two tailed	t=1.494	P=0.1858	
**Supp Fig.2D**		Unpaired t-test	two tailed	t=4.062	P=0.0066	
**Supp Fig.2I**	**oPSC Latency**	Unpaired t-test	two tailed	t=0.5213	P=0.6032	
**Supp Fig.2I**	**oEPSC Amplitude**	RM one-way ANOVA with Tukey's Post Hoc test		Treatment, F (2, 12) = 19.12	P=0.0002	aCSF vs. TTX (P=0.0001); aCSF vs. TTX+4AP (P=0.0063)
**Supp Fig.2I**	**oIPSC Amplitude**	RM one-way ANOVA with Tukey's Post Hoc test		Treatment, F (2, 8) = 7.054	P=0.0171	aCSF vs. TTX (P=0.0143)
**Supp Fig.2O**	**Whole neuronal area**	Unpaired t-test	two tailed	t=5.520	P<0.0001	
**Supp Fig.2O**	**Neurite length**	Unpaired t-test	two tailed	t=3.362	P=0.0015	
**Supp Fig.2O**	**Basal dendrite area**	Unpaired t-test	two tailed	t=0.7566	P=0.4530	
**Supp Fig.2O**	**# Basal dendritic trees**	Unpaired t-test	two tailed	t=3.376	P=0.0014	
**Supp Fig.2O**	**# Basal dendritic ends**	Unpaired t-test	two tailed	t=1.680	P=0.0992	
**Supp Fig.3D**	**Tone (0-10s)**	RM two-way ANOVA with Bonferroni's Post Hoc test		Cell Type Main Effect, F (2, 18) = 11.48	P=0.0006	**Conditioning day1:** Con/Foff vs. Con/Fon (p=0.0097); **Conditioning day1:** Con/Foff vs. Coff/Fon (p=0.0002); **Conditioning day2:** Con/Foff vs. Con/Fon (p=0.0015); **Conditioning day2:** Con/Foff vs. Coff/Fon (p<0.0001); **Conditioning day3:** Con/Foff vs. Con/Fon (p<0.0001); **Conditioning day3**: Con/Foff vs. Coff/Fon (p<0.0001).
				Days Main Effect, F (5, 90) = 21.50	P<0.0001	**Con/Foff:** Conditioning day1 vs. Extinction day1 (p<0.0001); **Con/Foff:** Conditioning day1 vs. Extinction Day2 (p=0.0002); **Con/Foff:** Conditioning day1 vs. Renewal (p=0.0001); **Con/Foff:** Conditioning day2 vs. Extinction day1 (p<0.0001); **Con/Foff:** Conditioning day2 vs. Extinction day2 (p<0.0001); **Con/Foff:** Conditioning day2 vs. Renewal (p<0.0001); **Con/Foff:** Conditioning day3 vs. Extinction day1 (p<0.0001); **Con/Foff:** Conditioning day3 vs. Extinction day2 (p<0.0001); **Con/Foff:** Conditioning day3 vs. Renewal (p<0.0001); **Con/Fon:** Conditioning day2 vs. Extinction day1 (p=0.0067); **Con/Fon:** Conditioning day2 vs. Extinction day2 (p=0.0098).
				Cell Type x Days interaction, F (10, 90) = 4.024	P=0.0001	
**Supp Fig.3D**	**Tone (10-28s)**	RM two-way ANOVA with Bonferroni's Post Hoc test		Cell Type Main Effect, F (2, 18) = 6.638	P=0.0069	**Conditioning day1:** Con/Foff vs. Con/Fon (p=0.0069); **Conditioning day2:** Con/Foff vs. Con/Fon (p=0.0027); **Conditioning day2:** Con/Foff vs. Coff/Fon (p<0.0001); **Conditioning day3:** Con/Foff vs. Con/Fon (p<0.0001); **Conditioning day3**: Con/Foff vs. Coff/Fon (p<0.0001).
				Days Main Effect, F (5, 90) = 20.07	P<0.0001	**Con/Foff:** Conditioning day1 vs. Conditioning day3 (p=0.0034); **Con/Foff:** Conditioning day1 vs. Extinction day1 (p=0.0001); **Con/Foff:** Conditioning day1 vs. Extinction Day2 (p=0.0015); **Con/Foff:** Conditioning day1 vs. Renewal (p=0.0077); **Con/Foff:** Conditioning day2 vs. Extinction day1 (p<0.0001); **Con/Foff:** Conditioning day2 vs. Extinction day2 (p<0.0001); **Con/Foff:** Conditioning day2 vs. Renewal (p<0.0001); **Con/Foff:** Conditioning day3 vs. Extinction day1 (p<0.0001); **Con/Foff:** Conditioning day3 vs. Extinction day2 (p<0.0001); **Con/Foff:** Conditioning day3 vs. Renewal (p<0.0001); **Con/Fon:** Conditioning day2 vs. Extinction day1 (p=0.0272).
				Cell Type x Days interaction, F (10, 90) = 5.955	P<0.0001	
**Supp Fig.3D**	**Footshock (28-33s)**	RM two-way ANOVA		Cell Type Main Effect, F (2, 18) = 1.984	P=0.1665	
				Days Main Effect, F (2, 36) = 0.4397	P=0.6476	
				Cell Type x Days interaction, F (4, 36) = 0.7836	P=0.5433	
**Supp Fig.3D**	**Post-footshock (33-50s)**	RM two-way ANOVA with Bonferroni's Post Hoc test		Cell Type Main Effect, F (2, 18) = 10.24	P=0.0011	**Conditioning day1:** Con/Foff vs. Con/Fon (p<0.0001); **Conditioning day1:** Con/Fon vs. Coff/Fon (p=0.0386); **Conditioning day2:** Con/Foff vs. Con/Fon (p=0.0049); **Conditioning day3:** Con/Foff vs. Con/Fon (p=0.0003); **Conditioning day3**: Con/Fon vs. Coff/Fon (p=0.0041).
				Days Main Effect, F (2, 36) = 0.04810	P=0.9531	
				Cell Type x Days interaction, F (4, 36) = 1.311	P=0.2844	
**Supp Fig.3E**	**Conditioning day1, Tone**	RM two-way ANOVA with Bonferroni's Post Hoc test		Group Main Effect, F (2, 19) = 1.081	P=0.3591	
				Trial Main Effect, F (9, 171) = 23.48	P<0.0001	
				Group x Trial interaction, F (18, 171) = 2.505	P=0.0012	**Tone4:** Coff/Fon vs. Con/Foff (p=0.006); **Tone7:** Coff/Fon vs. Con/Foff (p=0.0172)

Figure	Sub-figure	Statistical Test	One tailed or two tailed?	t, D, or F value	P value	If there is signifance, where the signifciance occurred (multiple comparison)?
**Supp Fig.3E**	**Conditioning day2, Tone**	RM two-way ANOVA		Group Main Effect, F (2, 19) = 3.198	P=0.0635	
				Trial Main Effect, F (9, 171) = 3.677	P=0.0003	
				Group x Trial interaction, F (18, 171) = 1.273	P=0.2112	
**Supp Fig.3E**	**Conditioning day3, Tone**	RM two-way ANOVA		Group Main Effect, F (2, 19) = 1.178	P=0.3295	
				Trial Main Effect, F (9, 171) = 1.378	P=0.2016	
				Group x Trial interaction, F (18, 171) = 0.8264	P=0.6675	
**Supp Fig.3E**	**Extinction day1, Tone**	RM two-way ANOVA		Group Main Effect, F (2, 19) = 2.904	P=0.0794	
				Trial Main Effect, F (9, 171) = 1.038	P=0.4121	
				Group x Trial interaction, F (18, 171) = 1.406	P=0.1338	
**Supp Fig.3E**	**Extinction day2, Tone**	RM two-way ANOVA		Group Main Effect, F (2, 19) = 2.346	P=0.1229	
				Trial Main Effect, F (9, 171) = 6.339	P<0.0001	
				Group x Trial interaction, F (18, 171) = 0.8439	P=0.6467	
**Supp Fig.3E**	**Renewal, Tone**	RM two-way ANOVA		Group Main Effect, F (2, 19) = 2.993	P=0.0742	
				Trial Main Effect, F (4, 76) = 3.927	P=0.0060	
				Group x Trial interaction, F (8, 76) = 0.8558	P=0.5573	
**Supp Fig.3E**	**Conditioning day1, ITI**	RM two-way ANOVA		Group Main Effect, F (2, 19) = 0.4370	P=0.6523	
				Trial Main Effect, F (9, 171) = 32.53	P<0.0001	
				Group x Trial interaction, F (18, 171) = 0.9072	P=0.5707	
**Supp Fig.3E**	**Conditioning day2, ITI**	RM two-way ANOVA		Group Main Effect, F (2, 19) = 2.790	P=0.0866	
				Trial Main Effect, F (9, 171) = 3.650	P=0.0003	
				Group x Trial interaction, F (18, 171) = 1.436	P=0.1204	
**Supp Fig.3E**	**Conditioning day3, ITI**	RM two-way ANOVA		Group Main Effect, F (2, 19) = 3.090	P=0.0689	
				Trial Main Effect, F (9, 171) = 0.6076	P=0.7895	
				Group x Trial interaction, F (18, 171) = 1.079	P=0.3768	
**Supp Fig.3E**	**Extinction day1, ITI**	RM two-way ANOVA		Group Main Effect, F (2, 19) = 1.581	P=0.2317	
				Trial Main Effect, F (9, 171) = 3.053	P=0.0020	
				Group x Trial interaction, F (18, 171) = 1.344	P=0.1663	
**Supp Fig.3E**	**Extinction day2, ITI**	RM two-way ANOVA with Bonferroni's Post Hoc test		Group Main Effect, F (2, 19) = 3.851	P=0.0394	**ITI2:** Con/Fon vs. Coff/Fon (p=0.0151); **ITI2:** Con/Fon vs. Con/Foff (p=0.0012); **ITI4:** Con/Fon vs. Con/Foff (p=0.0055); **ITI18:** Con/Fon vs. Con/Foff (p=0.0185); **ITI20:** Con/Fon vs. Con/Foff (p=0.0495)
				Trial Main Effect, F (9, 171) = 2.173	P=0.0261	
				Group x Trial interaction, F (18, 171) = 2.074	P=0.0086	
**Supp Fig.3E**	**Renewal, ITI**	RM two-way ANOVA		Group Main Effect, F (2, 19) = 1.220	P=0.3173	
				Trial Main Effect, F (4, 76) = 1.508	P=0.2082	
				Group x Trial interaction, F (8, 76) = 0.5764	P=0.7942	

Figure	Sub-figure	Statistical Test	One tailed or two tailed?	t, D, or F value	P value	If there is signifance, where the signifciance occurred (multiple comparison)?
**Supp Fig.4C**		RM two-way ANOVA		Tone Main Effect, F (4, 28) = 0.3200	P=0.8622	
				Days Main Effect, F (2, 14) = 0.6314	P=0.5463	
				Tone x Days interaction, F (8, 56) = 1.959	P=0.0690	
**Supp Fig.4D**		RM two-way ANOVA		Tone Main Effect, F (9, 63) = 1.775	P=0.0908	
				Days Main Effect, F (1, 7) = 0.2860	P=0.6093	
				Tone x Days interaction, F (9, 63) = 0.8102	P=0.6086	
**Supp Fig.4E**		RM one-way ANOVA with Tukey's Post Hoc test		Tone, F (4, 28) = 3.870	P=0.0126	Tone1 vs. Tone2 (P=0.0211); Tone1 vs. Tone3 (p=0.0200).
**Supp Fig.5B**	**Tone**	RM two-way ANOVA with Bonferroni's Post Hoc test		Trial Main Effect, F (5, 190) = 87.02	P<0.0001	
				Group Main Effect, F (2, 38) = 3.891	P=0.0290	PDyn-lox_GFP-Cre vs. WT_GFP-Cre (p=0.0304)
				Trial x Group interaction, F (10, 190) = 0.9070	P=0.5278	
**Supp Fig.5B**	**ITI**	RM two-way ANOVA with Bonferroni's Post Hoc test		Trial Main Effect, F (5, 190) = 81.99	P<0.0001	
				Group Main Effect, F (2, 38) = 6.096	P=0.0051	PDyn-lox_GFP-Cre vs. WT_GFP-Cre (p=0.0351); PDyn-lox_eGFP vs. WT_GFP-Cre (p=0.0055)
				Trial x Group interaction, F (10, 190) = 0.6769	P=0.7451	
**Supp Fig.5C**	**Context recall**	RM two-way ANOVA		Time Main Effect, F (4, 84) = 4.272	P=0.0034	
				shRNA Main Effect, F (1, 21) = 1.315	P=0.2645	
				Time x shRNA interaction, F (4, 84) = 1.922	P=0.1142	
**Supp Fig.5C**	**Extinction Tone**	RM two-way ANOVA		Trial Main Effect, F (4, 84) = 8.982	P<0.0001	
				shRNA Main Effect, F (1, 21) = 0.02167	P=0.8844	
				Trial x shRNA interaction, F (4, 84) = 0.9908	P=0.4172	
**Supp Fig.5C**	**Extinction ITI**	RM two-way ANOVA		Trial Main Effect, F (4, 84) = 22.04	P<0.0001	
				shRNA Main Effect, F (1, 21) = 0.9771	P=0.3342	
				Trial x shRNA interaction, F (4, 84) = 0.5602	P=0.6922	
**Supp Fig.5C**	**Renewal Tone**	RM two-way ANOVA		Trial Main Effect, F (4, 68) = 3.371	P=0.0141	
				shRNA Main Effect, F (1, 17) = 3.101	P=0.0962	
				Trial x shRNA interaction, F (4, 68) = 1.404	P=0.2418	
**Supp Fig.5C**	**Renewal ITI**	RM two-way ANOVA		Trial Main Effect, F (4, 68) = 6.740	P=0.0001	
				shRNA Main Effect, F (1, 17) = 0.1004	P=0.7552	
				Trial x shRNA interaction, F (4, 68) = 0.6114	P=0.6559	
**Supp Fig.5D**	**Freezing before conditioning**	RM two-way ANOVA		Cue Main Effect, F (1, 21) = 1.996	P=0.1724	
				shRNA Main Effect, F (1, 21) = 1.702	P=0.2061	
				Cue x shRNA interaction, F (1, 21) = 1.159	P=0.2939	
**Supp Fig.5D**	**Recall CS+**	RM two-way ANOVA		Trial Main Effect, F (2, 44) = 2.079	P=0.1372	
				shRNA Main Effect, F (1, 22) = 0.1810	P=0.6747	
				Trial x shRNA interaction, F (2, 44) = 2.575	P=0.0876	
**Supp Fig. 5D**	**Recall CS-**	RM two-way ANOVA		Trial Main Effect, F (2, 44) = 2.283	P=0.1139	
				shRNA Main Effect, F (1, 22) = 1.448	P=0.2416	
				Trial x shRNA interaction, F (2, 44) = 2.762	P=0.0741	
**Supp Fig. 5D**	**Recall discrimination index**	Unpaired t-test	two tailed	t=0.5406	P=0.5945	
**Supp Fig.5E**	**% time in center**	Unpaired t-test	two tailed	t=0.5794	P=0.5685	

Figure	Sub-figure	Statistical Test	One tailed or two tailed?	t, D, or F value	P value	If there is signifance, where the signifciance occurred (multiple comparison)?
**Supp Fig.5E**	**Distance**	RM two-way ANOVA		Zone Main Effect, F (1, 21) = 758.9	P<0.0001	
				shRNA Main Effect, F (1, 21) = 3.039	P=0.0959	
				Zone x shRNA interaction, F (1, 21) = 0.6209	P=0.4395	
**Supp Fig.5F**		Unpaired t-test	two tailed	t=1.233	P=0.2313	
**Supp Fig.5G**	**Time in open arm (%)**	Unpaired t-test	two tailed	t=0.4763	P=0.6388	
**Supp Fig.5G**	**Open arm entries**	Unpaired t-test	two tailed	t=0.1390	P=0.8908	
**Supp Fig.5G**	**Distance in open arm**	Unpaired t-test	two tailed	t=0.6522	P=0.5213	
**Supp Fig.5H**	**Air puff**	RM two-way ANOVA		Time Main Effect, F(2711,32532)=11.14	p<0.0001	
				shRNA Main Effect, F(1,12)=0.7521	p=0.4028	
				Time x shRNA interaction, F(2711,32532)=0.8162	p>0.9999	
**Supp Fig.5H**	**Tail shock**	RM two-way ANOVA		Time Main Effect, F(2711,29821)=10.57	p<0.0001	
				shRNA Main Effect, F(1,11)=0.3917	p=0.9298	
				Time x shRNA interaction, F(2711,29821)=0.8468	p>0.999	
**Supp Fig.5I**		RM two-way ANOVA		Test Main Effect, F (1, 21) = 2.343	P=0.1408	
				shRNA Main Effect, F (1, 21) = 0.007956	P=0.9298	
				Test x shRNA interaction, F (1, 21) = 0.1164	P=0.7363	
**Supp Fig.5J**		Unpaired t-test	two tailed	t=1.285	P=0.213	
**Supp Fig.5K**	**Time to respond**	Unpaired t-test	two tailed	t=0.2221	P=0.8258	
**Supp Fig.5K**	**Time to jump**	Unpaired t-test	two tailed	t=0.5450	P=0.5899	
**Supp Fig.5L**		RM two-way ANOVA with Bonferroni's Post Hoc test		Treatment Main Effect, F (1, 29) = 152.8	P<0.0001	**shCtrl:** pre-CFA vs. post-CFA (p<0.0001); **shPDyn:** pre-CFA vs. post-CFA (p<0.0001).
				shRNA Main Effect, F (1, 29) = 0.02259	P=0.8816	
				Treatment x shRNA interaction, F (1, 29) = 0.2013	P=0.6570	
**Supp Fig.5M**		RM two-way ANOVA with Bonferroni's Post Hoc test		Treatment Main Effect, F (1, 29) = 46.26	P<0.0001	**shCtrl:** pre-CFA vs. post-CFA (p<0.0001); **shPDyn:** pre-CFA vs. post-CFA (p=0.0011).
				shRNA Main Effect, F (1, 29) = 0.2041	P=0.6548	
				Treatment x shRNA interaction, F (1, 29) = 1.134	P=0.2957	
**Supp Fig.6A**	**shCtrl Trial 1**	Kolmogorov-Smirnov test		D=0.1085	P=0.0004	Tone 1 vs. Shock 1
				D=0.1864	P<0.0001	Tone 1 vs. ITI 1
				D=0.08345	P=0.0134	Shock 1 vs. ITI 1
**Supp Fig.6A**	**shPDyn Trial 1**	Kolmogorov-Smirnov test		D=0.1102	P<0.0001	Tone 1 vs. Shock 1
				D=0.1070	P<0.0001	Tone 1 vs. ITI 1
				D=0.1587	P<0.0001	Shock 1 vs. ITI 1
**Supp Fig.6A**	**shCtrl Trial 6**	Kolmogorov-Smirnov test		D=0.1335	P<0.0001	Tone 1 vs. Tone 6
				D=0.1544	P<0.0001	Tone 1 vs. Shock 6
				D=0.1446	P<0.0001	Tone 1 vs. ITI 6
				D=0.05007	P=0.3283	Tone 6 vs. Shock 6
				D=0.05563	P=0.2158	Tone 6 vs. ITI 6
				D=0.07371	P=0.0402	Shock 6 vs. ITI 6
**Supp Fig.6A**	**shPDyn Trial 6**	Kolmogorov-Smirnov test		D=0.08661	P=0.0010	Tone 1 vs. Tone 6
				D=0.1309	P<0.0001	Tone 1 vs. Shock 6
				D=0.06947	P=0.0148	Tone 1 vs. ITI 6
				D=0.06791	P=0.0184	Tone 6 vs. Shock 6
				D=0.06922	P=0.0153	Tone 6 vs. ITI 6
				D=0.1184	P<0.0001	Shock 6 vs. ITI 6
