# Supplementary material for: Prefrontal cortical dynorphin peptidergic transmission constrains threat-driven behavioral and network states
Source: bioRxiv. 2024 Jan 9:2024.01.08.574700. Preprint. [Version 1] doi: 10.1101/2024.01.08.574700 (PMC10822088; doi:10.1101/2024.01.08.574700)
Supplement: Supplement 2 [file media-2.pdf]

KEY RESOURCES TABLE

REAGENT or RESOURCE	SOURCE	IDENTIFIER
Antibodies		
Anti-ProDynorphin antibody	Abcam	Cat# ab10280
Anti-GFP antibody	Abcam	Cat# ab13970
Anti-Somatostatin Antibody, clone YC7	MilliporeSigma	Cat# MAB354
Alexa Fluor® 488 AffiniPure Donkey Anti-Guinea Pig IgG (H+L)	Jackson ImmunoResearch	Cat# 706-545-148
Alexa Fluor® 594 AffiniPure Donkey Anti-Guinea Pig IgG (H+L)	Jackson ImmunoResearch	Cat# 706-585-148
Alexa Fluor® 594 AffiniPure Donkey Anti-Rat IgG (H+L)	Jackson ImmunoResearch	Cat# 712-585-153
Goat Anti-Chicken IgY H&L (Alexa Fluor® 488) preabsorbed	Abcam	Cat# ab150173
Bacterial and Virus Strains		
AAV2/9-phSyn1(S)-Flex-tdTomato-T2A-SypEGFP-WPRE	Boston Children's Hospital Viral Core	NA
AAV1-Syn-FLEX-ChrimsonR-tdTomato	UNC vector core	Lot# AV6554B
AAVrg-EF1α-DIO-eYFP	Addgene	Cat# 27056
AAV5-EF1α-DIO-ChR2-eYFP	UNC vector core	Lot# AV4313-2A
AAVrg-FLEX-tdTomato	Addgene	Cat# 28306
AAV-Syn-Con/Foff-eYFP	UNC vector core	Lot# AV6151
AAV-Syn-Con/Fon-eYFP	UNC vector core	Lot# AV6148B
AAVrg-hDlx-Flex-GFP	Addgene	Cat# 83895
AAV-EF1α-DIO-eYFP	UNC vector core	Lot# AV4310K
AAV9-syn-FLEX-jGCaMP7f-WPRE	Addgene	Cat# 104492
AAV9-syn-jGCaMP7f-WPRE	Addgene	Car# 104488
AAV8-EF1a-Con/Fon-GCaMP6m	Dr. Karl Deisseroth, Stanford	NA
AAV8-EF1a-Coff/Fon-GCaMP6m	Dr. Karl Deisseroth, Stanford	NA
AAV8-EF1a-Con/Foff-GCaMP6m	Dr. Karl Deisseroth, Stanford	NA

AAV1-Syn-kLight 1.2	Dr. Lin Tian, UC Davis	NA
AAV1-Syn-kLight 1.3	Dr. Lin Tian, UC Davis	NA
AAV1-Syn-kLight 0	Dr. Lin Tian, UC Davis	NA
AAV5-U6-PDyn-shRNA-GFP	Custom packaged by Vigene	NA
AAV5-U6-scrambled-shRNA-GFP	Custom packaged by Vigene	NA
AAV5-U6-PDyn-shRNA-tdTomato	Custom packaged by Vigene	NA
AAV5-U6-scrambled-shRNA-tdTomato	Custom packaged by Vigene	NA
Chemicals, Peptides, and Recombinant Proteins		
(-)-U-50488 hydrochloride	Tocris	Cat# 0496
Naloxone hydrochloride	Tocris	Cat# 0599
Dyn A 1-17	NIDA	Cat# MPSP-015
Dyn A 2-17		
nor-Binaltorphimine dihydrochloride (nor-BNI)	Tocris	Cat# 0347
Tetrodotoxin citrate (TTX)	Tocris	Cat# 1069
4-Aminopyridine (4AP)	Tocris	Cat# 0940
Experimental Models: Organisms/Strains		
Mouse: WT: C57BL/6J	The Jackson Laboratory	Strain #:000664
Mouse: PDyn-Cre: B6;129S-*Pdyn*$^{tm1.1(cre)Mjkr}$/LowlJ	The Jackson Laboratory	Strain #:027958
Mouse: Ai14: B6.Cg-*Gt(ROSA)26Sor*$^{tm14(CAG-tdTomato)Hze}$/J	The Jackson Laboratory	Strain #:007914
Mouse: SST-FlpO: B6J.Cg-*Sst*$^{tm3.1(flpo)Zjh}$/AreckJ	The Jackson Laboratory	Strain #:031629
Oligonucleotides		
RNAscope™ Probe-Mm-Pdyn	Advanced Cell Diagnostics	Cat# 318771
RNAscope™ Probe-Mm-Slc17a7-C2	Advanced Cell Diagnostics	Cat# 416631-C2
RNAscope™ Probe-Mm-Slc32a1-C3	Advanced Cell Diagnostics	Cat# 319191-C3

RNAscope™ Probe-Mm-Sst-C2	Advanced Cell Diagnostics	Cat# 404631-C2
RNAscope™ Probe-Mm-Pvalb-C3	Advanced Cell Diagnostics	Cat# 421931-C3
RNAscope™ Probe- Hs-PDYN	Advanced Cell Diagnostics	Cat# 507161
RNAscope™ Probe- Hs-SLC17A7-C2	Advanced Cell Diagnostics	Cat# 415611-C2
RNAscope™ Probe- Hs-SLC32A1-C3	Advanced Cell Diagnostics	Cat# 415681-C3
Software and Algorithms		
Fiji (ImageJ)	Schneider et al., 2012	https://imagej.net/software/fiji/
GraphPad Prism 9	GraphPad software	https://www.graphpad.com/scientific-software/prism/
Clampex and Clampfit 11	Molecular Devices	https://www.moleculardevices.com/products/axon-patch-clamp-system
Adobe Illustrator	Adobe	https://www.adobe.com/products/illustrator.html
FreezeFrame 4 and FreezeFrame 5	Actimetrics	https://actimetrics.com/products/freezeframe/
TopScan	Clever Sys Inc.	http://cleversysinc.com/CleverSysInc/csi_products/topscan-suite/
ANY-maze	Stoelting	http://www.anymaze.co.uk/index.htm
Synapse	Tucker-Davis Technologies	https://www.tdt.com/component/synapse-software/
Inscopix Data Acquisition Software	Inscopix	https://www.inscopix.com/nvoke
Inscopix Data Processing Software	Inscopix	https://www.inscopix.com/nvoke
RStudio	RStudio	https://www.rstudio.com/
Python	Python Software Foundation	https://www.python.org/
Bonsai	Lopes et al., 2015	https://bonsai-rx.org/
