# Supplementary material for: Prefrontal cortical dynorphin peptidergic transmission constrains threat-driven behavioral and network states
Source: bioRxiv. 2024 Jan 9:2024.01.08.574700. Preprint. [Version 1] doi: 10.1101/2024.01.08.574700 (PMC10822088; doi:10.1101/2024.01.08.574700)
Supplement: 3 [file NIHPP2024.01.08.574700v1-supplement-3.pdf]

bioRxiv preprint doi: https://doi.org/10.1101/2024.01.08.574700; this version posted January 9, 2024. The copyright holder for this preprint (which was not certified by peer review) is the author/funder. This article is a US Government work. It is not subject to copyright under 17 USC 105 and is also made available for use under a CC0 license.

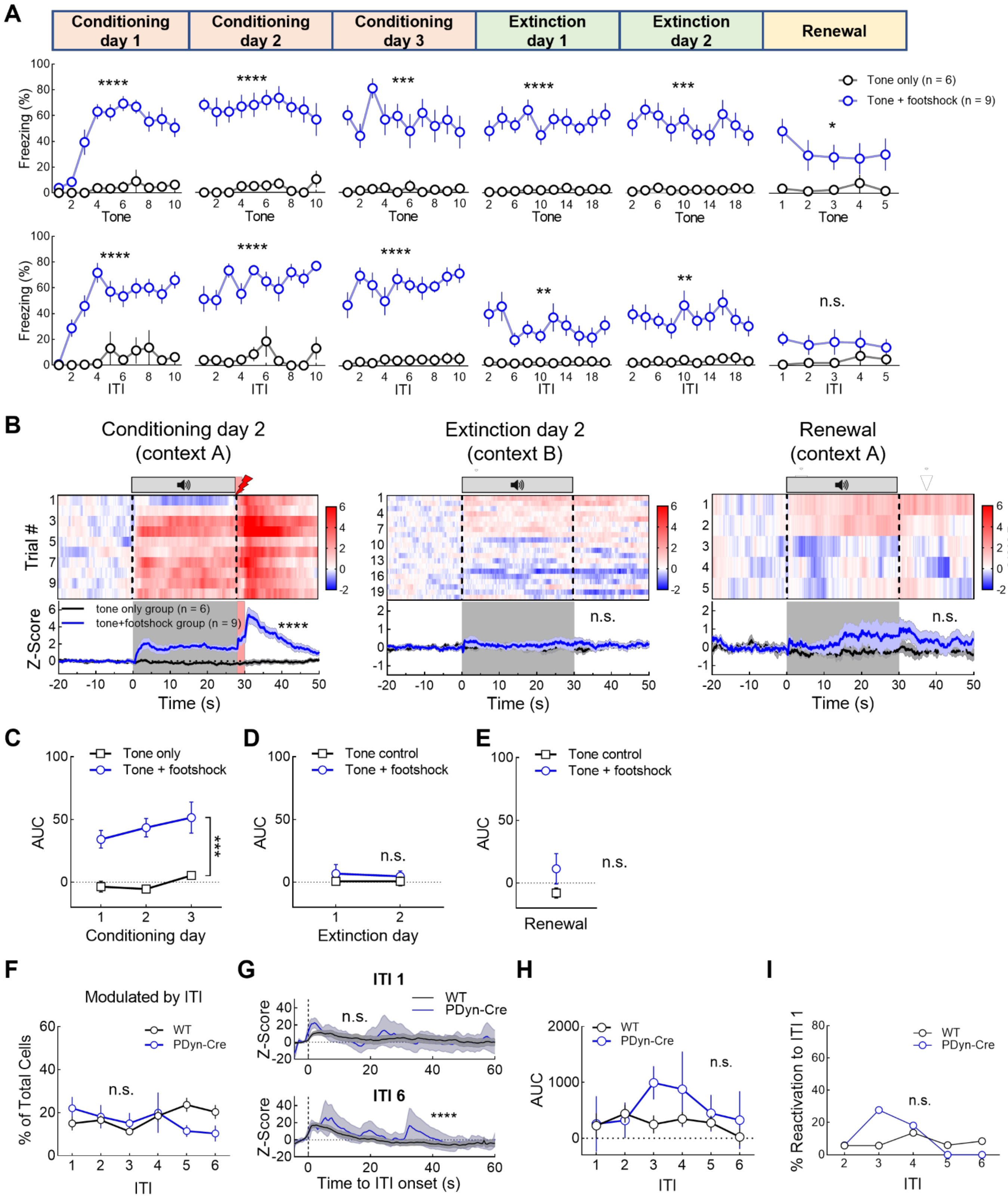

Figure S1: Relevant to Figure 1 In-vivo monitoring of mPFC^{Dyn} cells during fear conditioning

bioRxiv preprint doi: https://doi.org/10.1101/2024.01.08.574700; this version posted January 9, 2024. The copyright holder for this preprint (which was not certified by peer review) is the author/funder. This article is a US Government work. It is not subject to copyright under 17 USC 105 and is also made available for use under a CC0 license.

A) Freezing during tone and ITI period across threat conditioning, threat recall/extinction day 1 and extinction day 2, and renewal. Data is binned every 2 trials for extinction days (See Statistics Table for statistics).

B) Heatmaps of fiber photometry GCaMP activity across the session during day 2 of threat conditioning, day 2 of threat extinction, and renewal of threat responsivity in the training context after extinction (RM two-way ANOVA, group main effect ****$p<0.0001$).

C-E) AUC analysis of Ca^{2+} activity evoked during conditioning (C), extinction (D), and renewal (E) (Two-way ANOVA for C and D, group main effect, ***p=0.0006; unpaired t-test for E).

F) Percentage of neurons significantly modulated by the intertrial interval (Two-way ANOVA, cell type main effect, p=0.6202).

G) Ca^{2+} responses aligned to ITI onset in intertrial interval-modulated neurons (Two-way ANOVA, cell type main effect, ****p<0.0001).

H) AUC of Ca^{2+} responses during the intertrial intervals (Two-way ANOVA, cell type main effect, p=0.0734).

I) The percentage of footshock encoding neurons during intertrial interval 1 that were also significantly activated during all subsequent intertrial intervals (paired t-test, p=0.6738).

bioRxiv preprint doi: https://doi.org/10.1101/2024.01.08.574700; this version posted January 9, 2024. The copyright holder for this preprint (which was not certified by peer review) is the author/funder. This article is a US Government work. It is not subject to copyright under 17 USC 105 and is also made available for use under a CC0 license.

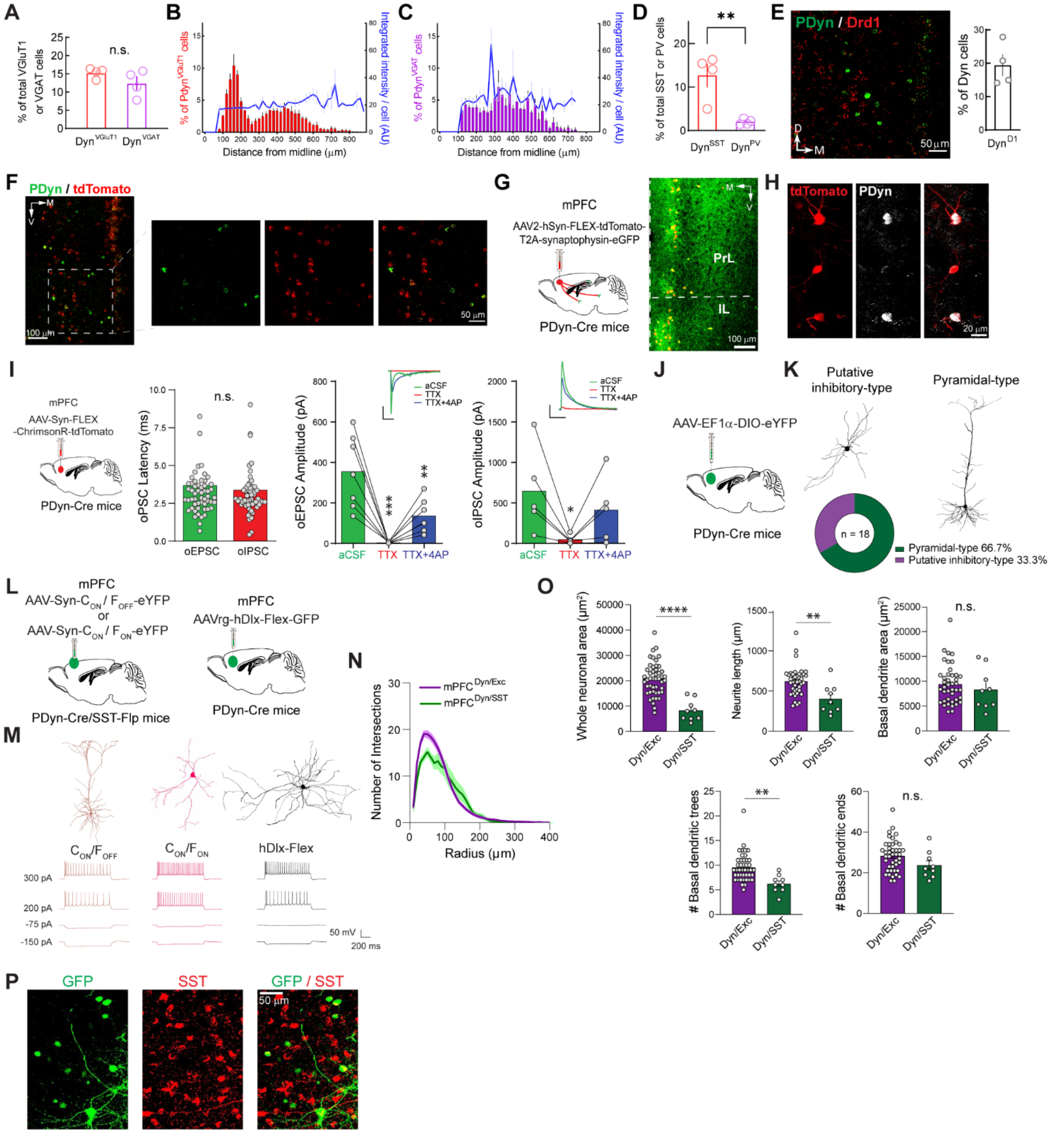

Figure S2: Relevant to Figure 2 Anatomical characterization of mPFC dynorphin-expressing neurons

bioRxiv preprint doi: https://doi.org/10.1101/2024.01.08.574700; this version posted January 9, 2024. The copyright holder for this preprint (which was not certified by peer review) is the author/funder. This article is a US Government work. It is not subject to copyright under 17 USC 105 and is also made available for use under a CC0 license.

A) The proportion of PDyn mRNA-containing neurons does not differ between mPFC excitatory and inhibitory neuron populations (unpaired t-test, *p*=0.1858).

B) Percentage of VGluT1-positive mPFC^{Dyn} neurons and their levels of PDyn mRNA expression across the medial-lateral gradient of the mPFC.

C) Same as above but for VGAT-positive neurons that co-express PDyn mRNA.

D) Percentage of mPFC SST- and PV-positive interneurons that co-express PDyn mRNA (unpaired t-test, ***p*=0.0066).

E) Representative image of PDyn (green) and Drd1a (red) mRNA expression in the mPFC of WT mice. The bar graph shows quantification of the co-expression of Drd1a mRNA in mPFC^{Dyn} cells.

F) Tomato-positive cells from PDyn-iCre mice crossed with Ai14 tdTomato reporter mice do not reliably label cells that express PDyn mRNA during adulthood. Representative confocal image of 20X RNAscope in-situ hybridization showing that PDyn mRNA (green) is largely absent in the majority of tdTomato mRNA-positive cells (red), suggesting ectopic expression of tdTomato.

G) Schematic and representative image of AAV-hSyn-FLEX-tdTomato-T2A-Synaptophysin-eGFP expression in the mPFC of PDyn-Cre mice.

H) PDyn immunoreactivity in mPFC neurons expressing tdTomato-2A-Synaptophysin-eGFP in a Cre-dependent manner.

I) Mean onset latency of mPFC^{Dyn} neurons evoked oEPSCs and oIPSCs consistent with direct monosynaptic excitatory and inhibitory connections, respectively (unpaired t-test, *p*=0.6032). The monosynaptic connections were also confirmed by TTX and 4-AP application (**p*=0.0143, ***p*=0.0063, ****p*=0.0001).

J) Schematic AAV-EF1α-DIO-eYFP expression in the mPFC of PDyn-Cre mice.

K) Representative reconstructions of mPFC^{Dyn} cells exhibiting pyramidal and non-pyramidal morphologies indicate putative excitatory and inhibitory neurons, respectively. Pie chart shows percentage of pyramidal and putative inhibitory mPFC^{Dyn} cells.

L) INTRSECT and hDlx promoter approaches to label mPFC$^{Dyn/SST}$ neurons.

M) Non-pyramidal mPFC$^{Dyn/SST}$ cells labeled with C_{ON}/F_{ON} INTRSECT and hDlx promoter approaches and excitatory pyramidal neurons labeled with C_{ON}/F_{OFF}

bioRxiv preprint doi: https://doi.org/10.1101/2024.01.08.574700; this version posted January 9, 2024. The copyright holder for this preprint (which was not certified by peer review) is the author/funder. This article is a US Government work. It is not subject to copyright under 17 USC 105 and is also made available for use under a CC0 license.

INTRSECT. I/V curve in current clamp recordings from these $mPFC^{Dyn/SST}$ and $mPFC^{Dyn/Exc}$ cells.

N) Sholl analysis of $mPFC^{Dyn/SST}$ neuron morphology.

O) Summary of whole neuronal area, neurite length, basal dendrite area, number of basal dendritic trees, and number of dendritic ends of $mPFC^{Dyn/Exc}$ and $mPFC^{Dyn/SST}$ cells (***p*<0.01, *****p*<0.0001).

P) Additional representative images of SST immunoreactivity (red) on hDlx-Flex-GFP expression (green) mPFC cells as in Fig. 2L.

bioRxiv preprint doi: https://doi.org/10.1101/2024.01.08.574700; this version posted January 9, 2024. The copyright holder for this preprint (which was not certified by peer review) is the author/funder. This article is a US Government work. It is not subject to copyright under 17 USC 105 and is also made available for use under a CC0 license.

1

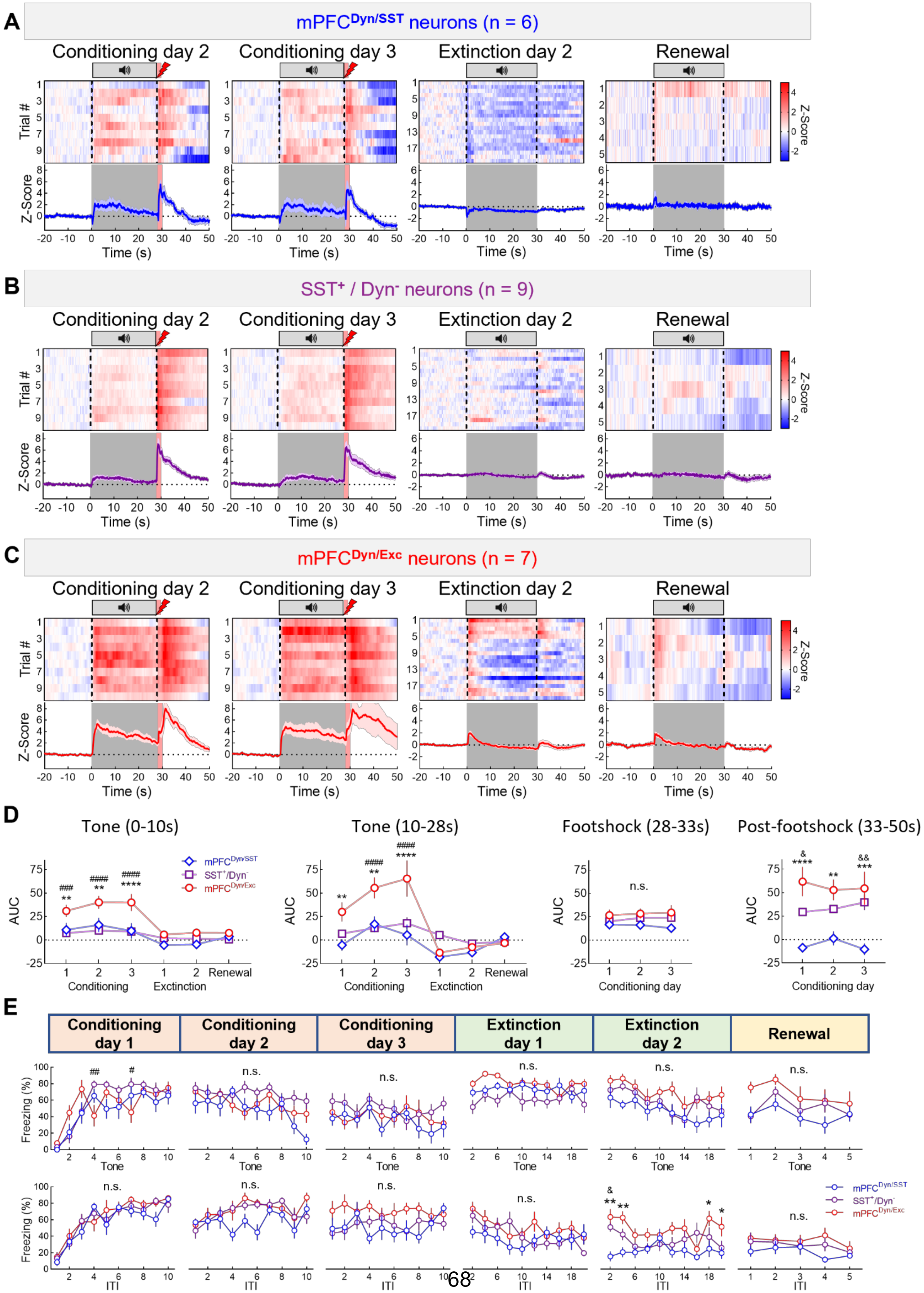

bioRxiv preprint doi: https://doi.org/10.1101/2024.01.08.574700; this version posted January 9, 2024. The copyright holder for this preprint (which was not certified by peer review) is the author/funder. This article is a US Government work. It is not subject to copyright under 17 USC 105 and is also made available for use under a CC0 license.

Figure S3: Relevant to Figure 3 Activity of excitatory and inhibitory vmPFC^{Dyn} cells during threat conditioning, extinction and renewal

A) Heatmaps and traces of GCaMP activity in mPFC$^{Dyn/SST}$ neurons across the session during day 2 and day 3 of threat conditioning, day 2 of threat extinction, and renewal of threat responsitivity in the training context after extinction.

B) Heatmaps and traces of GCaMP activity in SST^{+} /Dyn^{-} neurons across the session during day 2 and day 3 of threat conditioning, day 2 of threat extinction, and renewal of threat responsitivity in the training context after extinction.

C) Heatmaps and traces of GCaMP activity in mPFC$^{Dyn/Exc}$ neurons across the session during day 2 and day 3 of threat conditioning, day 2 of threat extinction, and renewal of threat responsitivity in the training context after extinction.

D) AUC of Ca^{2+} activity during early tone (0-10s, Two-way ANOVA, cell-type main effect with Bonferroni's post-hoc test, $^{**}p<0.01$ $^{****}p<0.0001$ between mPFC$^{Dyn/SST}$ and mPFC$^{Dyn/Exc}$ neurons, $^{\#\#\#}p<0.001$ $^{\#\#\#\#}p<0.0001$ between SST^{+}/Dyn^{-} and mPFC$^{Dyn/Exc}$ neurons), late tone (10-28s, Two-way ANOVA, cell-type main effect with Bonferroni's post-hoc test, $^{**}p<0.01$ $^{****}p<0.0001$ between mPFC$^{Dyn/SST}$ and mPFC$^{Dyn/Exc}$ neurons, $^{\#\#\#\#}p<0.0001$ between SST^{+}/Dyn^{-} and mPFC$^{Dyn/Exc}$ neurons), footshock (28-33s, Two-way ANOVA), and post footshock periods (33-50s, Two-way ANOVA, cell-type main effect with Bonferroni's post-hoc test, $^{**}p=0.0049$ $^{***}p=0.0003$ $^{****}p<0.0001$ between mPFC$^{Dyn/SST}$ and mPFC$^{Dyn/Exc}$ neurons, $^{\&}p=0.0386$ $^{\&\&}p=0.0041$ between mPFC$^{Dyn/SST}$ and SST^{+}/Dyn^{-} neurons).

E) Freezing during tone and ITI period across threat conditioning, threat recall/extinction day 1 and extinction day 2, and renewal in 3 INTRSECT groups of mice. Data is binned every 2 trials for extinction days (Two-way ANOVA, $^{*}p<0.05$ $^{**}p<0.01$ between mPFC$^{Dyn/SST}$ and mPFC$^{Dyn/Exc}$ group, $^{\#}p=0.0172$ $^{\#\#}p=0.006$ between SST^{+}/Dyn^{-} and mPFC$^{Dyn/Exc}$ group, $^{\&}p=0.0151$ between mPFC$^{Dyn/SST}$ and SST^{+}/Dyn^{-} group).

bioRxiv preprint doi: https://doi.org/10.1101/2024.01.08.574700; this version posted January 9, 2024. The copyright holder for this preprint (which was not certified by peer review) is the author/funder. This article is a US Government work. It is not subject to copyright under 17 USC 105 and is also made available for use under a CC0 license.

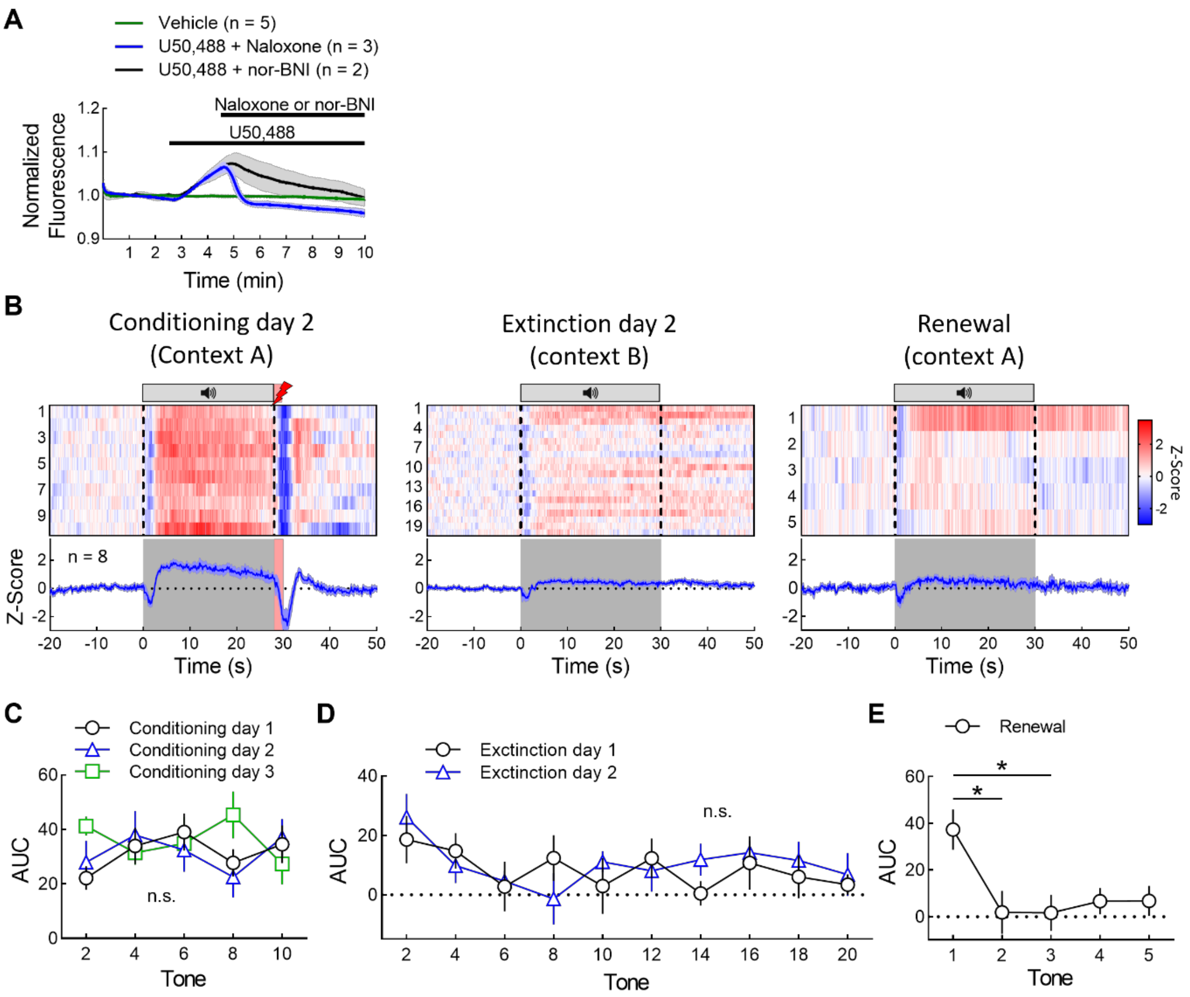

Figure S4: Relevant to Figure 4 In-vivo monitoring of putative/ dynorphin/KOR signaling

A) Naloxone or nor-BNI blocks increase of mPFC kLight1.2 fluorescence by U50,488 in slices.

B) Heatmaps and traces of kLight activity in mPFC across the session during day 2 of threat conditioning, day 2 of threat extinction, and renewal of threat responsitivity.

C-E) AUC of kLight signal during threat conditioning (C), threat extinction (D), and renewal of threat responsitivity (E). Data is binned every 2 trials in C and D (Two-way ANOVA for C and D, ANOVA with Tukey's Post Hoc test for E, *p<0.05).

bioRxiv preprint doi: https://doi.org/10.1101/2024.01.08.574700; this version posted January 9, 2024. The copyright holder for this preprint (which was not certified by peer review) is the author/funder. This article is a US Government work. It is not subject to copyright under 17 USC 105 and is also made available for use under a CC0 license.

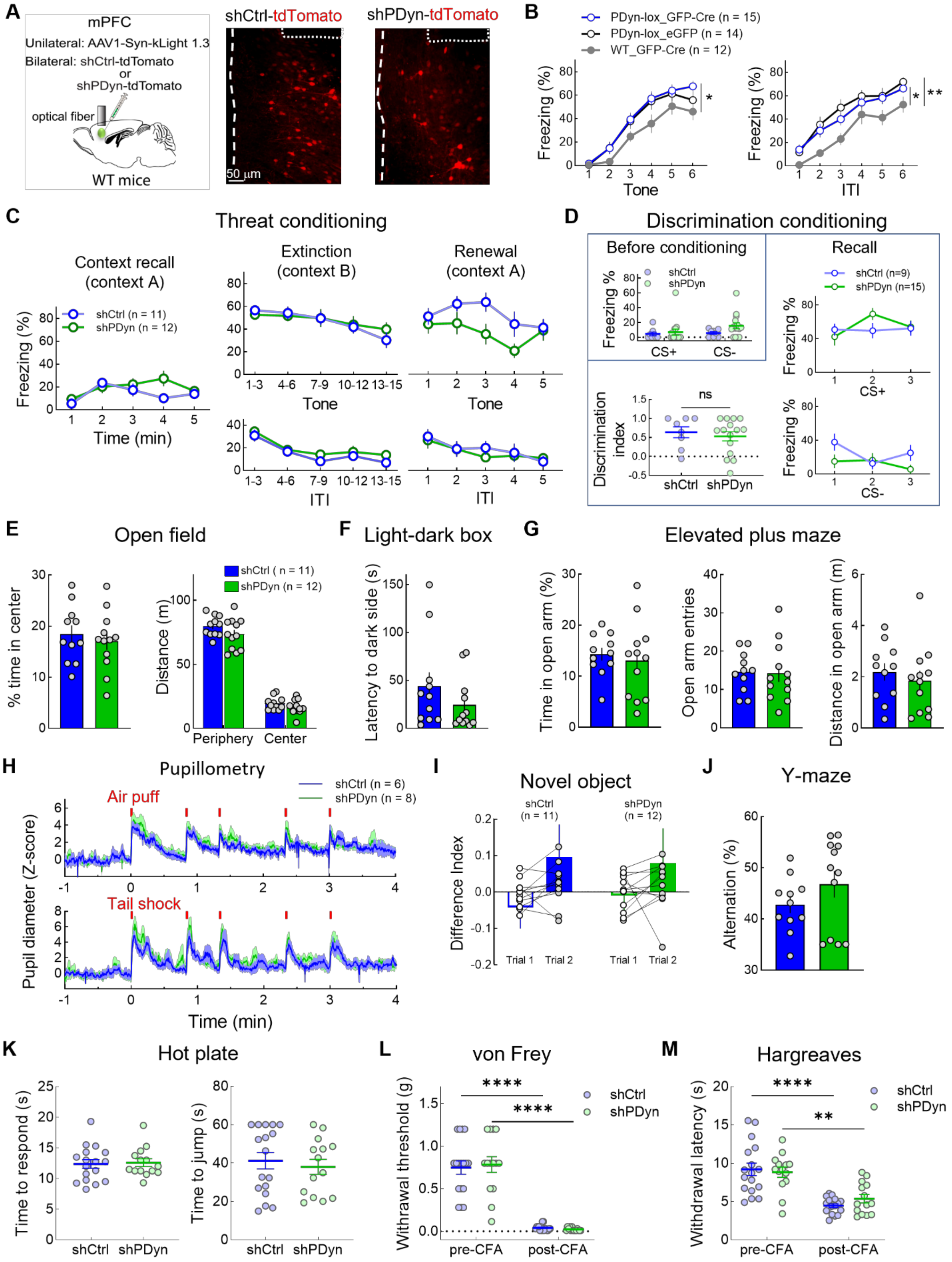

1

bioRxiv preprint doi: https://doi.org/10.1101/2024.01.08.574700; this version posted January 9, 2024. The copyright holder for this preprint (which was not certified by peer review) is the author/funder. This article is a US Government work. It is not subject to copyright under 17 USC 105 and is also made available for use under a CC0 license.

Figure S5: Relevant to Figure 5 PDyn knockdown in the vmPFC does not impact on recall or extinction of fear memory, recall of discriminative cues, locomotion, anxiety, autonomic response, and working memory.

A) Representative images of shCtrl-tdTomato or shPDyn-tdTomato expression in WT mice of kLight fiber photometry recording.

B) Freezing during tone and ITI in PDyn-loxP mice expressing AAV-Cre-GFP or AAV-eGFP, and in WT mice expressing AAV-Cre-GFP in the vmPFC (Two-way ANOVA with Bonferroni's Post Hoc test, Tone: *p=0.0304 between PDyn-lox_GFP-Cre and WT_GFP-Cre; ITI: *p=0.0351 between PDyn-lox_GFP-Cre and WT_GFP-Cre, **p=0.0055 between PDyn-lox_eGFP and WT_GFP-Cre).

C) Contextual threat recall, cued threat recall/extinction, and renewal of conditioned freezing.

D) vmPFC Dyn knockdown does not modify basal freezing during the baseline of the cue discrimination task or during the cued recall of the CS+ and CS-.

E) vmPFC PDyn does not regulate time spent in the center of the open field or total distance moved in the periphery or center of the arena.

F) Latency to the dark side in the light dark-box test.

G) No significant difference in elevated plus maze test.

H) No significant differences in air-puff or shock-evoked pupil dilation.

I) No significant difference in novel object recognition memory.

J) No significant differences in working memory as assessed by the spontaneous alternation task in the Y-maze.

K) No significant difference in time to respond and time to jump at the hot plate test.

L) No significant difference in von Frey test before or after CFA treatment (Two-way ANOVA with Bonferroni's Post Hoc test, Treatment Main Effect, ****p<0.0001).

M) No significant difference in Hargreaves test before or after CFA treatment (Two-way ANOVA with Bonferroni's Post Hoc test, Treatment Main Effect, **p=0.0011, ****p<0.0001).

Same group of mice were used in Fig. 5F,H as in Fig. S5C, E-G, and I,J.

bioRxiv preprint doi: https://doi.org/10.1101/2024.01.08.574700; this version posted January 9, 2024. The copyright holder for this preprint (which was not certified by peer review) is the author/funder. This article is a US Government work. It is not subject to copyright under 17 USC 105 and is also made available for use under a CC0 license.

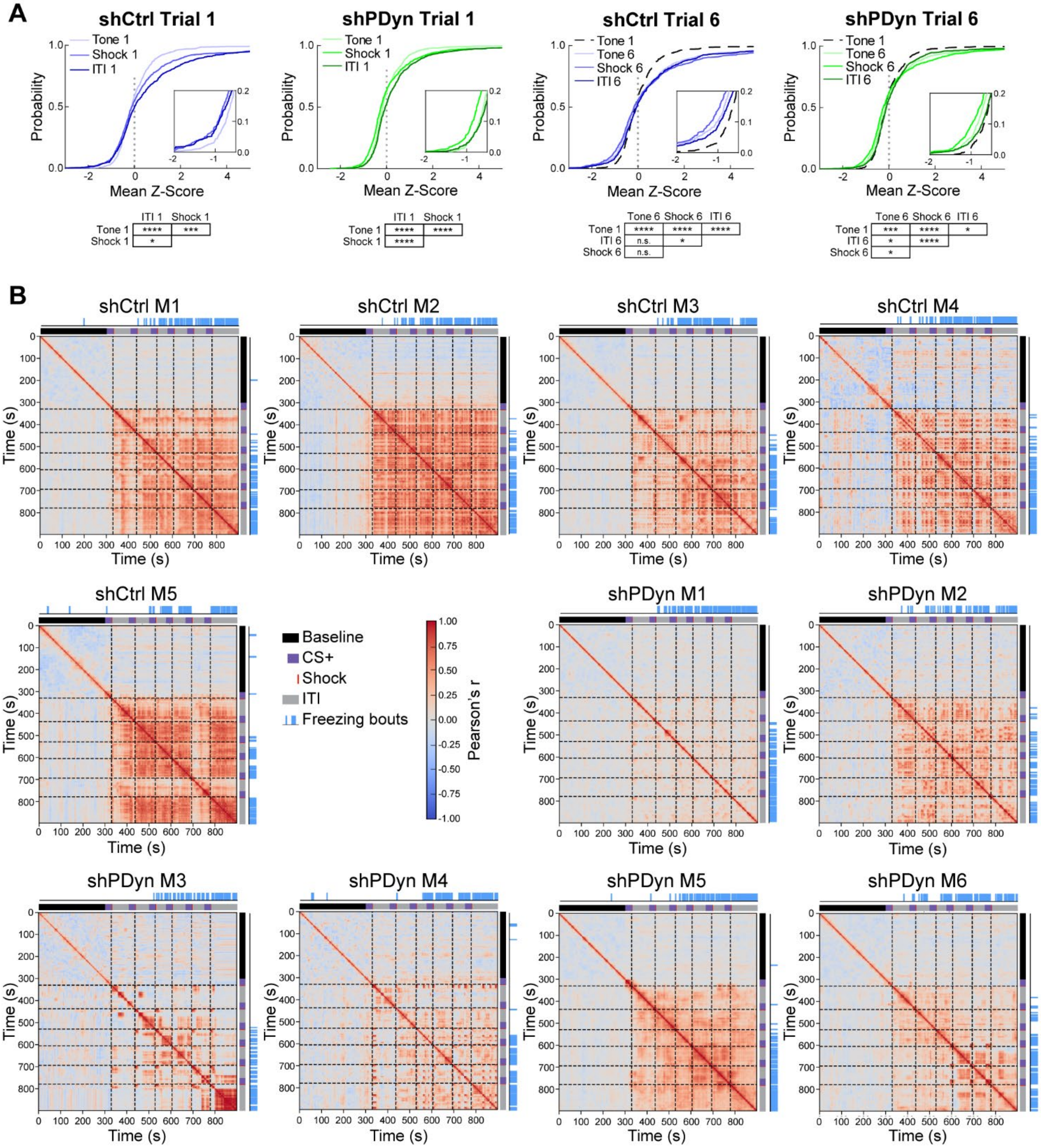

Figure S6: Relevant to Figure 6 and Figure 7. PCA trajectory and PCC data in individual mice

A) Cumulative probability of mean Z-Score in control (blue) and PDyn-shRNA (green) mice during the Tone periods, shock periods, and ITI from trial 1 and trial 6. Insets

bioRxiv preprint doi: https://doi.org/10.1101/2024.01.08.574700; this version posted January 9, 2024. The copyright holder for this preprint (which was not certified by peer review) is the author/funder. This article is a US Government work. It is not subject to copyright under 17 USC 105 and is also made available for use under a CC0 license.

are zoomed in between Mean Z-score -2 to -0.5. Statistical significance from pair-wise Kolmogorov-Smirnov test is shown under each plot (*$p<0.05$, ***$p<0.001$, ****$p<0.0001$).

B) Correlation matrices from individual mice in control-shRNA and PDyn-shRNA groups overlayed with freezing bouts.
